# Genetic Basis of Increased Lifespan and Postponed Senescence in *Drosophila melanogaster*

**DOI:** 10.1534/g3.120.401041

**Published:** 2020-01-22

**Authors:** Grace A. Parker, Nathan Kohn, Ally Spirina, Anna McMillen, Wen Huang, Trudy F. C. Mackay

**Affiliations:** * Department of Biological Sciences,; † Program in Genetics,; ‡ W. M. Keck Center for Behavioral Biology, North Carolina State University, Raleigh, North Carolina, 27695 and; § Department of Animal Science, Michigan State University, East Lansing, Michigan, 48824

**Keywords:** Laboratory evolution, candidate genes, RNAi

## Abstract

Limited lifespan and senescence are near-universal phenomena. These quantitative traits exhibit variation in natural populations due to the segregation of many interacting loci and from environmental effects. Due to the complexity of the genetic control of lifespan and senescence, our understanding of the genetic basis of variation in these traits is incomplete. Here, we analyzed the pattern of genetic divergence between long-lived (O) *Drosophila melanogaster* lines selected for postponed reproductive senescence and unselected control (B) lines. We quantified the productivity of the O and B lines and found that reproductive senescence is maternally controlled. We therefore chose 57 candidate genes that are expressed in ovaries, 49 of which have human orthologs, and assessed the effects of RNA interference in ovaries and accessary glands on lifespan and reproduction. All but one candidate gene affected at least one life history trait in one sex or productivity week. In addition, 23 genes had antagonistic pleiotropic effects on lifespan and productivity. Identifying evolutionarily conserved genes affecting increased lifespan and delayed reproductive senescence is the first step toward understanding the evolutionary forces that maintain segregating variation at these loci in nature and may provide potential targets for therapeutic intervention to delay senescence while increasing lifespan.

Limited lifespan and senescence, the post-reproductive decline in survival and fertility with advancing age, are near-universal phenomena. They are quantitative traits that exhibit variation in natural populations due to the segregation of many different interacting loci and environmental effects (Finch and Tanzi 1997; Finch and Ruvkun 2001; Pasyukova *et al.* 2004; Pitt and Kaeberlein 2015).

Due to the complexity of the genetic control of lifespan and senescence, our understanding of the genetic basis of variation in these traits is incomplete. Evolutionary theory predicts that variants affecting lifespan may have antagonistic effects on other aspects of fitness (Williams 1957), have late-life specific deleterious effects (Medawar 1952) and/or have negative pleiotropic effects on reproduction and somatic maintenance (Kirkwood 1977), explaining why genetic variation for lifespan may persist. Studies in *Drosophila melanogaster* provide experimental support for these predictions as there is increased genetic variance in mortality (Hughes and Charlesworth 1994; Charlesworth and Hughes 1996) and fecundity (Durham *et al.* 2014) with increasing age; negative genetic correlations between early fecundity and lifespan (Rose and Charlesworth 1981a) and reduced early fecundity and increased lifespan of lines selected for late-age fecundity (Rose and Charlesworth 1981b; Rose 1984; Luckinbill *et al.* 1984; Sgrò and Partridge 1999; Remolina *et al.* 2012; Fabian *et al.* 2018), and single mutations affecting increased lifespan have deleterious effects on other fitness-related quantitative traits (Magwire *et al.* 2010). However, to date only a few causal genes underlying these relationships in a natural population have been identified (Paaby and Schmidt 2008; Paaby *et al.* 2014). Identifying specific genes with allelic variants that causally affect lifespan and senescence will enhance our understanding of the evolutionary forces acting on these genes and empirically test the validity and relative contributions of the evolutionary theories of senescence and maintenance of genetic variation. These causal genes may also offer potential targets for therapeutic intervention to delay senescence while increasing lifespan.

Many mechanisms influencing lifespan have been implicated by studies of effects of mutations and segregating natural variation in short-lived model organisms and humans, such as insulin signaling (Friedman and Johnson 1988; Kenyon *et al.* 1993; Kimura *et al.* 1997; Paradis and Ruvkun 1998; Tissenbaum and Ruvkun 1998; Gil *et al.* 1999; Clancy *et al.* 2001; Holzenberger *et al.* 2003; Blüher *et al.* 2003; Giannakou *et al.* 2004; Hwangbo *et al.* 2004; Paaby *et al.* 2014), caloric restriction (Lakowski and Hekimi 1998; Defossez *et al.* 2001; Lin *et al.* 2002; Rogina and Helfand 2004; Grandison *et al.* 2009), environmental stress (Rose *et al.* 1992; Lithgow *et al.* 1995; Zwaan *et al.* 1995; Lin *et al.* 1998; Mockett and Sohal 2006; Rollmann *et al.* 2006; Ma *et al.* 2015), DNA repair and replication (Woodhead *et al.* 1985; Yu *et al.* 1996; de Boer *et al.* 2002), telomere integrity (Bodnar *et al.* 1998), immune response (Zerofsky *et al.* 2005; Felix *et al.* 2012; Horn *et al.* 2014), sensory perception (Apfeld and Kenyon 1999; Libert *et al.* 2007), gene silencing (Kim *et al.* 1999), learning (Ping *et al.* 2015), and reactive oxygen species (ROS) detoxification (Griswold *et al.* 1993; Ishii *et al.* 1998; Parkes *et al.* 1998; Sun *et al.* 2002; Kharade *et al.* 2005). While many mutations have been identified that extend lifespan, many more decrease longevity, suggesting that normal expression of the latter genes is essential for survival. For example, a screen for *P*-element insertions affecting lifespan in *D. melanogaster* identified 135 genes associated with an increase in lifespan and 296 genes associated with a decrease in lifespan (Magwire *et al.* 2010).

Quantitative trait loci (QTL) affecting lifespan have been mapped in *D. melanogaster* (Nuzhdin *et al.* 1997; Leips and Mackay 2000; Pasyukova *et al.* 2000; Vieira *et al.* 2000; Leips and Mackay 2002; Forbes *et al.* 2004; Wilson, Morgan, and Mackay 2006; Durham *et al.* 2014; Ivanov *et al.* 2015; Fabian *et al.* 2018; Huang *et al.* 2020), *C. elegans* Shook 1996; Ayyadevara *et al.* 2001; Shmookler Reis *et al.* 2006), mice (Jackson *et al.* 2002; Doria *et al.* 2004; Lang *et al.* 2010), and humans (Beekman *et al.* 2013; Deelen *et al.* 2013; Deelen *et al.* 2014). However, only a few genes implicated by QTL mapping have been shown to be capable of affecting both lifespan and reproductive senescence using an independent assay (Durham *et al.* 2014).

Here, we used genetic divergence between five long-lived (O) *D. melanogaster* lines selected for postponed reproductive senescence and five unselected control (B) lines (Rose 1984; Carnes *et al.* 2015) to identify candidate genes underlying the response to selection. The O lines live twice as long as the B lines and maintain high levels of reproduction at later ages compared to the B lines (Carnes *et al.* 2015). We re-analyzed the genetic divergence data for these lines (Carnes *et al.* 2015) to identify intervals containing multiple variants with allele frequency differences between the O and B lines. These regions contained a large number of genes that could not be further resolved with existing genetic data. We then evaluated the effect of RNAi for 57 of these candidate genes on lifespan of males and females as well as lifetime reproductive success. We identified candidate genes for which RNAi increased lifespan and decreased early reproduction and vice versa, candidate genes for which RNAi had opposite effects on lifespan in males and females, candidate genes for which RNAi that increased lifespan with no deleterious effects on reproduction, and one candidate gene for which RNAi increased both lifespan and reproduction. These results provide support for the antagonistic pleiotropy theory of aging (Williams 1957) and the basis for further analysis of causal polymorphic variants contributing to the response to selection.

## Materials and Methods

### Drosophila stocks

The five long-lived O lines have been maintained with 70-day generation intervals to preserve selection for delayed reproduction, and the five unselected B lines have been maintained with 14-day generation intervals (Rose 1984; Carnes *et al.* 2015). All RNAi lines were obtained from the Vienna Drosophila Resource Center. The 39 *P{KK}* RNAi lines are from the same genetic background, contain upstream activating sequence *UAS*-RNAi constructs for each candidate gene at the same locus on the second chromosome, and have no known off-target effects on other loci (Dietzl *et al.* 2007). The 18 *P{GD}* RNAi lines are from the same genetic background, contain *P*-element based transgenes in a random insertion site, and have no known off-target effects on other loci (Dietzl *et al.* 2007). All candidate genes tested are listed in Supplementary Table 4. *GAL4*-c825 is specific to ovaries and accessory glands. Apart from the O populations, which were maintained in cages at room temperature when not being used for assays, all stocks and experimental flies were maintained at 25°, 60–75% relative humidity, and on a 12-hour light-dark cycle.

### Productivity assays: O and B lines

We assessed the productivity of females from the five O (O1 – O5) and five B (B1 – B5) lines crossed to males from their own lines, and productivity of O and B females crossed to males from the other selection lines (O1♀×B1♂, O2♀×B2♂, O3♀×B3♂, O4♀×B4♂, O5♀×B5♂, B1♀×O1♂, B2♀×O2♂, B3♀×O3♂, B4♀×O4♂, B5♀×O5♂) as described in Carnes *et al.* (2015) with a few exceptions. Experimental flies were produced by allowing six males and six females to mate and lay eggs for three days in vials containing 5 mL culture medium. Offspring from these vials were collected on the day of eclosion, anesthetized using CO_2_, sorted into vials with three males or three females, and given 24 hr to recover before setting up the experimental vials. Three male and three female flies of each genotype were placed in each of 15 replicate vials and aged to three to five days old. They were then allowed to lay eggs for 24 hr on 5 mL culture media once a week for four weeks. The total number of adults from each vial was counted until day 16 post-eclosion and divided by the number of living females in that vial to give an average per female per vial. Experimental flies were transferred without anesthesia to new vials containing 2 mL culture media every 1-3 days to minimize bacterial and fungal infections. Data were analyzed by an analysis of variance (ANOVA) with the model *Y* = *µ* + *Gm + Gf* + *W* + *Gm*×*W* + *Gf*×*W* + *Gm*×*Gf + Gm*×*Gf*×*W* + *ε*, where *Gm* is the fixed effect of the mother’s genotype, *Gf* is the fixed effect of the father’s genotype, *W* is the fixed effect of week, and *ε* is the error, using JMP Pro 14 (SAS Institute, Cary, NC). Reduced model ANOVAs of form *Y* = *µ* + *Gm + Gf* + *Gm*×*Gf* + *ε* were also run for each week, and Tukey’s tests were performed to determine significant differences between each cross at each week.

### Identification of intervals with high genomic divergence

To identify genomic intervals that contained fixed or nearly fixed variants between the O and B lines, we first identified variants with allele frequency differences greater than 0.8 between the O and B lines. Next, we merged any adjacent variants whose allele frequency differences were greater than 0.8. These intervals were often interrupted by small numbers of variants that did not meet this stringent allele frequency difference threshold. These interruptions were merged with their flanking intervals if they contained fewer than three variants and if the minimum allele frequency differences were greater than 0.5. We calculated both the length in bp of these merged intervals (Figure 2) and the number of variants they contained (Supplemental Table 1). The interval length was computed as the distance between neighboring variants and tends to be an underestimate when the number of variants was small.

### Productivity and lifespan assays: RNAi lines

The first generation of experimental flies were produced by allowing six *GAL4*-c825 males and six *P{KK}* or *P{GD} UAS*-RNAi females to mate and lay eggs in vials containing 10 mL culture medium for two days. The *GAL4*-c825 driver is expressed in amniosera, adult female ovary, and adult male accessory glands and seminal vesicles. Two control genotypes were also used: F_1_ progeny of v60100, which is the isogenic strain in which the *P{KK} UAS*-RNAi lines were constructed with the empty *PhiC31* vector, crossed to *GAL4*-c825; and F_1_ progeny of v60000, which is the isogenic strain in which the *P{GD} UAS*-RNAi lines were constructed with a modified *pUAST* vector pMF3, crossed to *GAL4*-c825. Of the F_1_ progeny from each mating, 3 males and 3 females were sorted into vials containing 5 mL culture medium with 48 replicates for each genotype. Three females per vial were allowed to lay eggs for 24 hr on 10 mL culture media once a week for their entire lifespan, and the average number of F_2_ offspring per female in each vial was recorded until 16 days after the initial egg laying for 13 of the 48 replicates. Flies were transferred without anesthesia to new vials containing 5 mL culture media every 1-3 days to provide fresh food and minimize bacterial and fungal infections. Dead flies were removed, and their deaths recorded. The sum of the average number of offspring across all weeks determined the lifetime productivity for each vial. The mixed factorial ANOVA model *Y* = *µ* + *S* + *G* + *S*×*G* + *Rep*(*G*) + *S*×*Rep*(*G*) + *ε* was used to partition variation in lifespan between the fixed main effects of Sex (*S*), Genotype (*G*, RNAi *vs.* control) and their interaction, and the random effect of replicate vial (*Rep*), nested within genotype. Reduced model ANOVAs of form *Y* = *µ* + *G* + *Rep*(*G*) + *ε* were also run for males and females separately. The full fixed effect factorial ANOVA model for lifetime productivity, which is the summation of all individual weeks, was *Y* = *µ* + *G* + *ε*; where *G* is Genotype. The full fixed effect factorial ANOVA model for weekly productivity was *Y* = *µ* + *W* + *G* + *W*×*G* + *ε*; where *W* is Week and *G* is Genotype. Reduced models of form *Y* = *µ* + *G* + *ε* were run for each week. These analyses were performed in six blocks with different genes in each block.

### Gene expression analyses

The magnitude of RNAi-mediated suppression of gene expression was assessed using quantitative PCR (qPCR) of female ovaries and male accessory glands for a subset of the RNAi and control genotypes. Ovary tissue was dissected from females and accessory glands were dissected from males at 3-5 days old, with 10 ovaries and 10 accessory glands in each of two biological replicates per genotype, and RNA was extracted. cDNA was synthesized from 120 ng of total RNA using the iScript cDNA synthesis kit (Bio-Rad). We performed qPCR for each of three technical replicates per biological replicate using Maxima SYBR Green (Thermo Scientific). GAPDH was the internal control. The levels of expression for each gene were normalized against the internal control and compared to the RNAi line control to determine differences in gene expression of the RNAi mutants. We first assessed overall differences in gene expression using the ANOVA model Y = μ + L + G + L×G + Rep(*L×G*) *+ ε*, where *L* indicates the RNAi or control line, *G* is the gene tested, *R* is biological replicate, nested within line and gene, and *ε* is the residual (technical replicate) variance. Reduced models of form *Y* = *µ* + L + Rep(*L*) + *ε* were run for each gene. Effect and *p*-Value summaries were performed to determine significant differences of the RNAi line from the control line. All analyses were performed using JMP Pro 14 (SAS Institute, Cary, NC).

### Data availability

The DNA sequencing raw data are publicly available on GEO under SRA Project ID PRJNA286855. Supplementary Table 1 gives the raw data for the four week productivity assay of the pure and reciprocal corsses between the O and B lines. Supplementary Table 2 gives the analyses of variance (ANOVAs) of O and B line productivity. Supplementary Table 3 shows the genomic regions containing genetically divergent SNPS. Supplementary Table 4 gives the list of 57 candidate genes tested, including their FlyBase ID, gene name, gene, symbol, and the Vienna stock number used in the RNAi assay. Supplementary Table 5 gives the raw lifespan data for *GAL4-c825* × *UAS* RNAi and *GAL4-c825* × control F1 flies. Supplementary Table 6 gives the raw lifetime productivity data for *GAL4-c825* × *UAS* RNAi and *GAL4-c825* × control F1 flies. Supplementary Table 7 gives the raw weekly productivity data for *GAL4-c825* × *UAS* RNAi and *GAL4-c825* × control F1 flies. Supplementary Table 8 gives the analyses of variance (ANOVAs) of lifespan for *GAL4*-c825 *× UAS* RNAi and *GAL4*-c825 × control F1 flies. Supplementary Table 9 gives the analyses of variance (ANOVAs) of lifetime productivity for *GAL4*-c825 *× UAS* RNAi and *GAL4*-c825 × control F1 flies. Supplementary Table 10 gives the analyses of variance (ANOVAs) of weekly productivity for *GAL4*-c825 *× UAS* RNAi and *GAL4*-c825 × control F1 flies. Supplementary Table 11 gives the analyses of variance (ANOVAs) of ovary qPCR for *GAL4*-c825 *× UAS* RNAi and *GAL4*-c825 × control F1 flies. Supplementary Table 12 gives the analyses of variance (ANOVAs) of accessory gland qPCR for *GAL4*-c825 *× UAS* RNAi and *GAL4*-c825 × control F1 flies. Supplementary Table 13 is a summary of genes for which RNAi affects lifespan and/or productivity. Supplementary Figure 1 shows the results of ovary qPCR for the difference in expression between *GAL4*-c825 *× UAS* RNAi and *GAL4*-c825 × control F1 flies. Supplementary Figure 2 show the results of accessory gland qPCR for the difference in expression between *GAL4*-c825 *× UAS* RNAi and *GAL4*-c825 × control F1 flies.. Supplemental material available at figshare: https://doi.org/10.25387/g3.11503278.

## Results

### Maternal effects on productivity in the O and B lines

**Figure 1:**
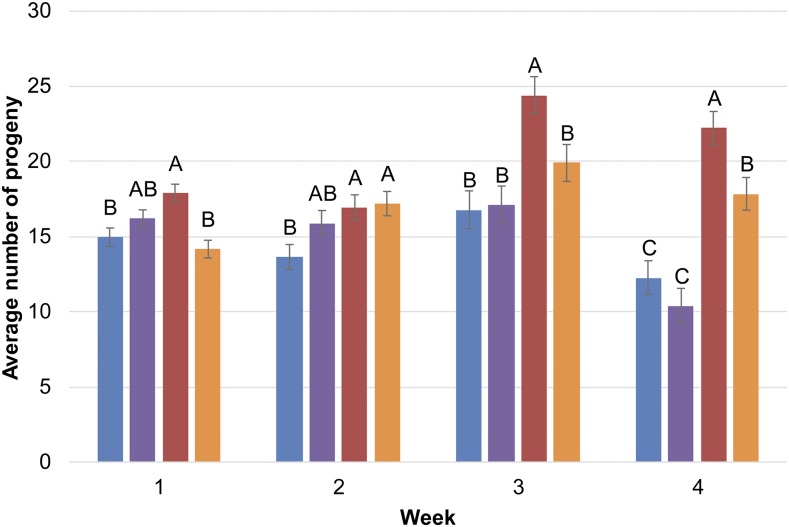
Average number of offspring per week. Blue bars: B♀×B♂. Purple bars: B♀×O♂. Red bars: O♀×B♂. Orange bars: O♀×O♂. Error bars are ± 1 SE. Letters denote significant differences at *P* < 0.05.

In order to assess whether the net effects of selection on reproductive senescence were maternally or paternally controlled, we quantified productivity for females of each of the O and B lines at one, two, three and four weeks of age. We assessed productivity for O females crossed to males of the same O line, O females crossed to B males; B females crossed to males from the same B line, and B females crossed to O males. We found little difference in the total number of viable offspring between any of these crosses for young flies, but divergence between the O and B genotypes at later ages (Figure 1, Supplementary Tables 1, 2). The B line females exhibited lower reproduction by week four than O line females, but no differences depending on the male genotype. In the cross with an O female and B male, there was higher productivity at week four than the cross of the O female and O male. These data indicate that productivity is largely maternally controlled, although the overall analysis does indicate significant effects of the week by male genotype (*P* = 0.012) and female genotype by male genotype (*P* = 0.013) interaction.

### Genomic intervals containing candidate genes

**Figure 2:**
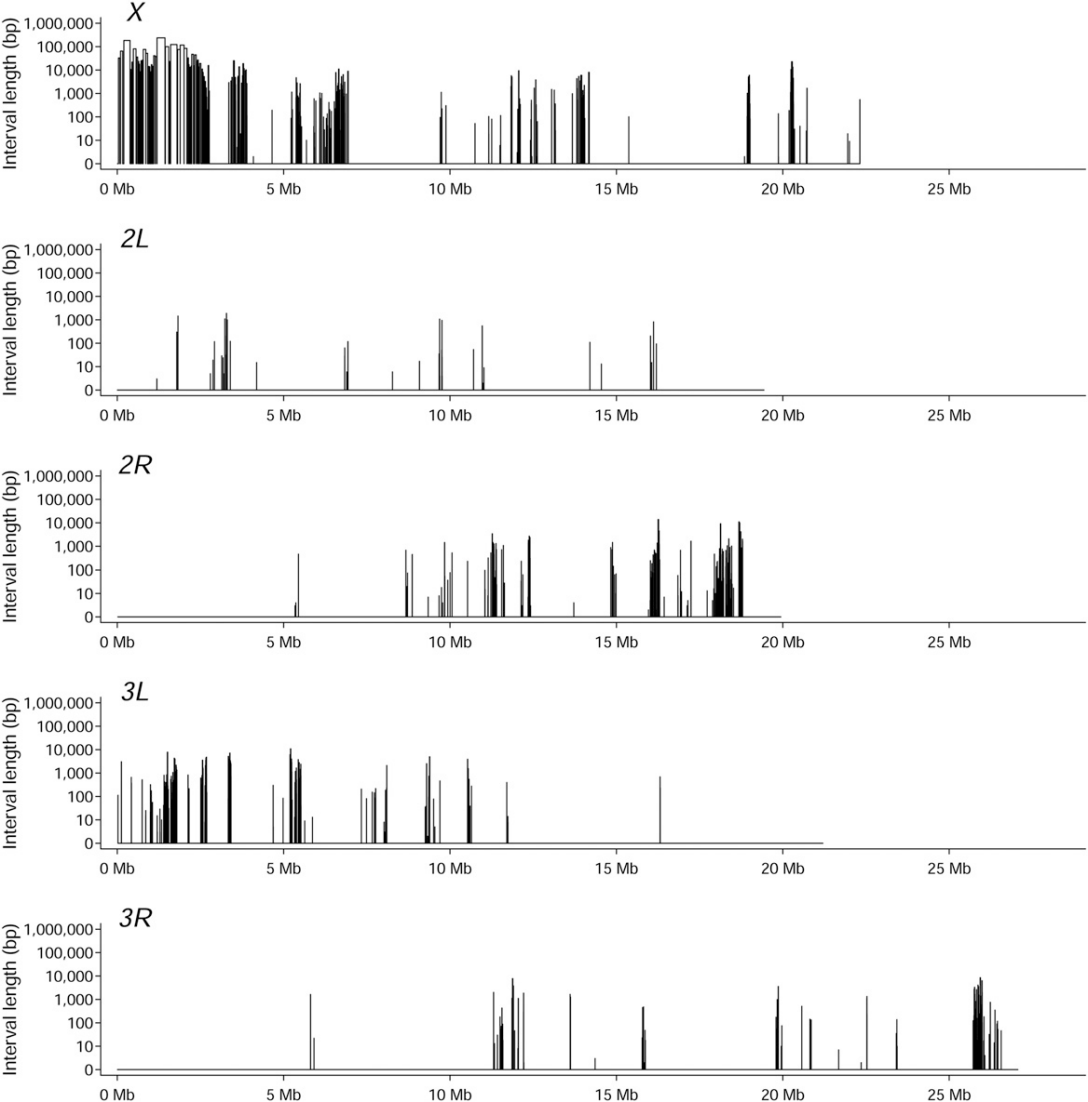
Analysis of intervals with high genomic divergence between the O and B lines. Each vertical bar indicates the locations of intervals with high genomic divergence on the major chromosome arms (*x*-axis) and the approximate length of each interval (bp) (*y*-axis).

Carnes *et al.* (Carnes *et al.* 2015) sequenced the genomes and transcriptomes of the O and B lines and assessed the genetic divergence between them, identifying 6,394 single nucleotide polymorphisms (SNPs) in or near 1,925 genes with nominally significant (*P* < 10^−3^) allele frequency differences, nearly 300 of which showed gene expression changes consistent with delayed senescence between young and old flies of the O and B lines. There were some large regions of genetic divergence between the O and B lines in this study, especially on the *X* chromosome. We re-analyzed these data, imposing the more stringent criterion that genetically divergent variants were those for which the average difference in allele frequency between the O and B lines was greater than 0.8. This analysis identified individual variants as well as intervals, some of which were quite large, containing multiple genetically divergent SNPs (Figure 2, Supplementary Table 3). The large genetically divergent intervals may have arisen as a consequence of selective sweeps, and Figure 2 presumably contain causal variants as well as linked non-causal loci that were driven to fixation or near fixation under selection for delayed reproductive senescence in the O lines relative to the B lines. Existing genetic data cannot resolve these regions any further. Therefore, it is necessary to assess the effects of each candidate gene on lifespan and reproduction in order to infer which ones are likely to harbor one or more functional polymorphisms responding to selection.

### Effects of RNAi on lifespan and productivity

We chose 57 candidate genes (Supplementary Table 4) that were either located in the intervals with high SNP divergence between the O and B lines (Supplementary Table 3), or that had low *P*-values from the previous genetic divergence analysis (Carnes *et al.* 2015) but for which the average allele frequency difference between the O and B lines was less than 0.8. The following criteria were used to choose among the many genes in the high divergence intervals: (1) the *P*-value for individual SNP divergence (Carnes *et al.* 2015) is < 10^−5^; (2) the candidate gene is expressed in ovaries and accessory glands since the productivity assay of the O and B lines implicated a strong maternal and weaker paternal effect, and selection was on reproductive senescence; and (3) viable RNAi stocks with no off-target effects were available. We assessed lifespan (Supplementary Table 5), average lifetime reproduction (Supplementary Table 6), and average weekly reproduction (Supplementary Table 7) for the F1 progeny of *UAS*-RNAi lines crossed to *GAL4*-c825, and F1 progeny of the crosses of an appropriate control line without the *UAS* transgene and the *GAL4* driver. *GAL4*-c825 is expressed in amniosera, adult female ovary, and adult male accessory glands and seminal vesicles and is therefore expected to reduce gene expression specifically in these tissues.

**Figure 3:**
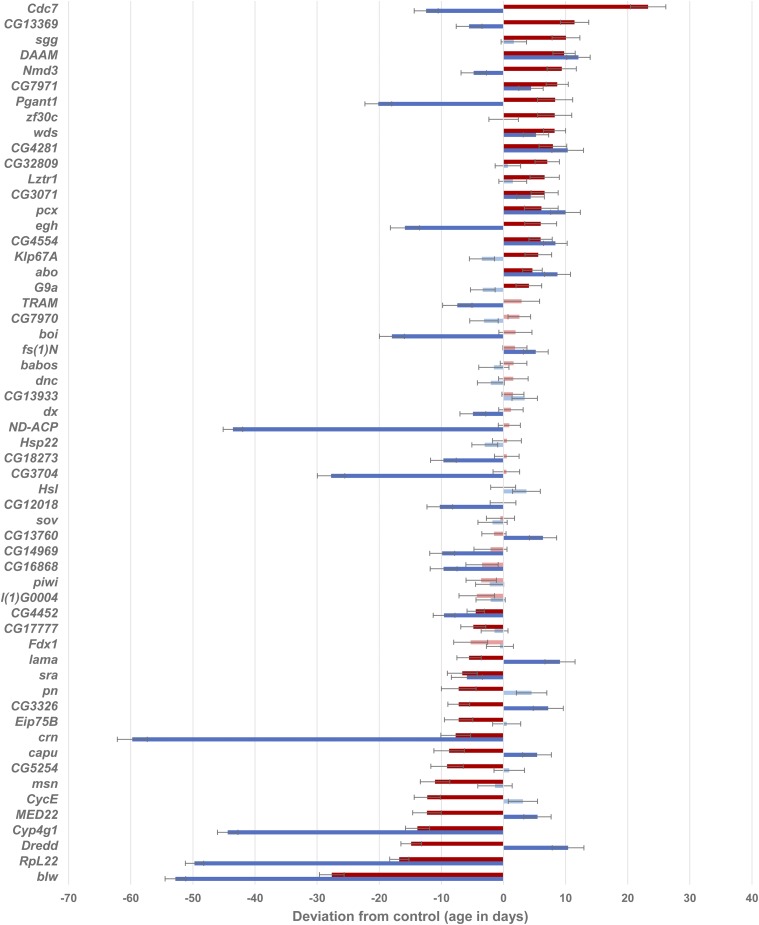
Differences in lifespan between *GAL4*-c825 × *UAS* RNAi and *GAL4*-c825 × control F1 flies. Error bars are ± 1 SE. Significance of the difference from control (*P* < 0.05) is denoted by darker colors. Red bars: Females. Blue bars: Males.

RNAi of 47 of the 57 genes tested affected lifespan (Figure 3, Supplementary Table 8). RNAi of 26 genes increased lifespan and 31 genes decreased lifespan in at least one sex; 10 of these genes had sexually antagonistic effects on lifespan (*capu*, *Cdc7*, *CG13369*, *CG3326*, *Dredd*, *egh*, *lama*, *MED22*, *Nmd3*, *Pgant1*). The effects on lifespan were highly sex-specific: seven genes increased lifespan in both sexes and six decreased lifespan in both sexes; eight genes increased lifespan in only one sex and 15 decreased lifespan in only one sex.

**Figure 4:**
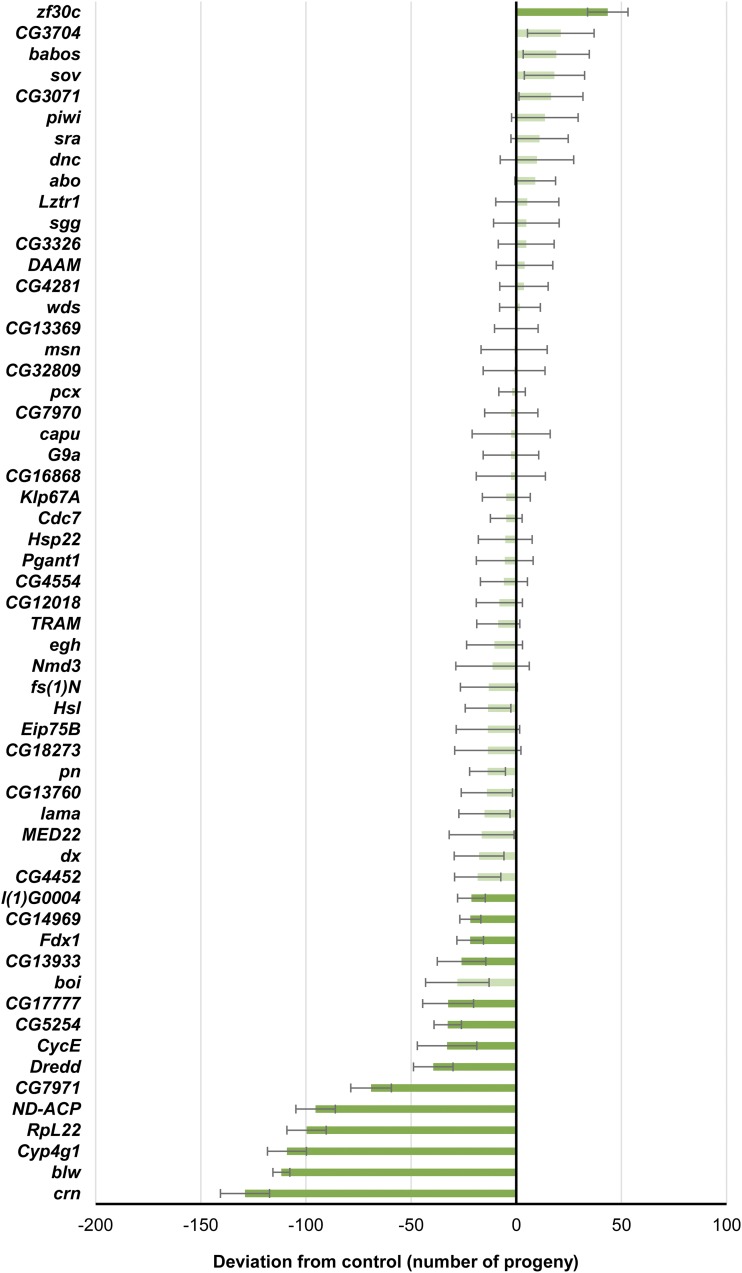
Differences in lifetime productivity (number of adult offspring) between *GAL4*-c825 *× UAS* RNAi and *GAL4*-c825 × control F1 flies. Error bars are ± 1 SE. Significance of the difference from control (*P* < 0.05) is denoted by darker colors.

**Figure 5:**
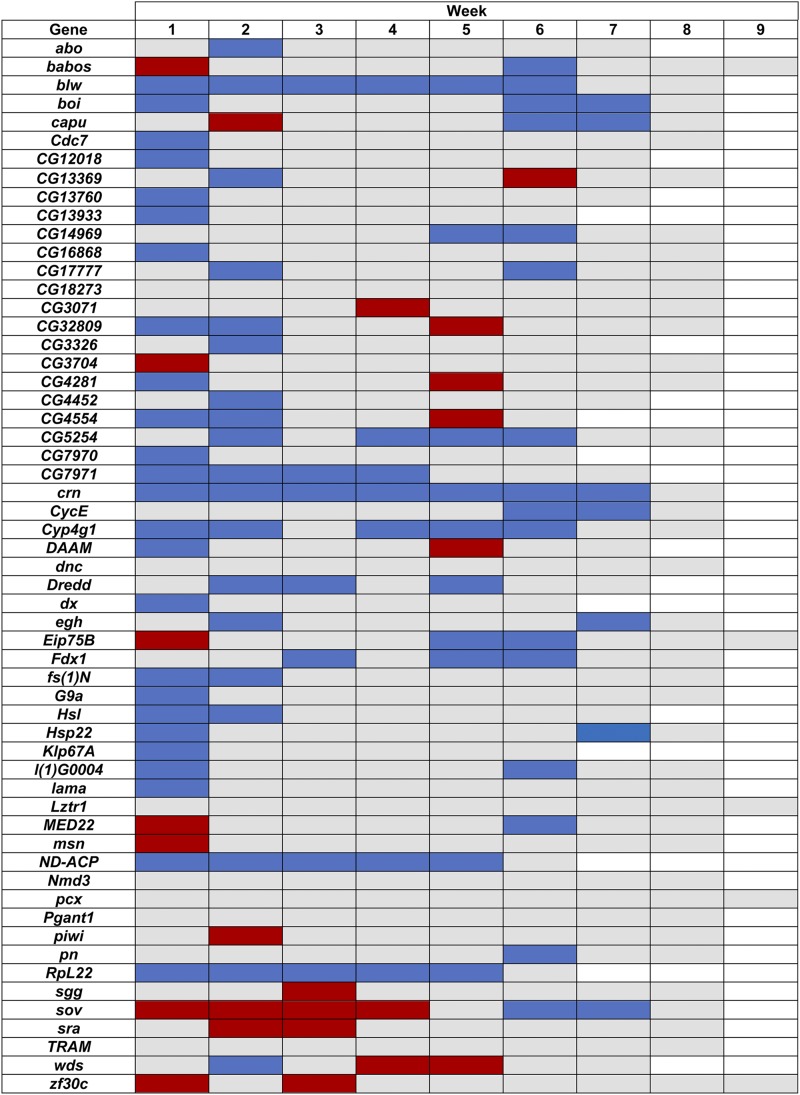
Significant differences (*P* < 0.05) in weekly productivity (number of adult offspring) between *GAL4*-c825 *× UAS* RNAi and *GAL4*-c825 × control F1 flies. Blue cells: Decreased productivity relative to the control. Red cells: Increased productivity relative to the control. Gray cells: Not significantly different from the control. White cells: No data due to decreased lifespan of *GAL4*-c825 × *UAS*-RNAi F1 flies.

RNAi of 15 genes affected total lifetime productivity: *zf30c* RNAi increased total productivity, while *blw*, *CG13933*, *CG14969*, *CG17777*, *CG5254*, *CG7971*, *crn*, *CycE*, *Cyp4g1*, *Dredd*, *Fdx1*, *l(1)G0004*, *ND-ACP* and *RpL22* RNAi decreased total productivity (Figure 4, Supplementary Table 9). However, RNAi of 50 candidate genes affected productivity in at least one week (Figure 5, Supplementary Table 10) and RNAi of 18 genes increased productivity in at least one week. Several genes appeared to have a tradeoff in reproduction. RNAi of *babos*, *capu*, *Eip75B*, *MED22*, and *sov* increased productivity at an early age and decreased productivity at later ages. RNAi of *CG13369*, *CG32809*, *CG4281*, *CG4554*, *DAAM*, and *wds* lowered productivity at an early age and increased productivity at later ages.

Given these data on lifespan and productivity, we can now assess the extent to which RNAi of these candidate genes gives the antagonistic pleiotropic effects between different fitness traits predicted by evolutionary theory (Williams 1957). RNAi of six genes affects lifespan and not productivity (Figure 3, Figure 5): *Lztr1*, *Nmd3*, *pcx* and *Pgant1* increase female lifespan with no deleterious effect on productivity; while *CG18273* and *TRAM* decrease male lifespan but do not have significant effects on productivity. RNAi of nine genes affects productivity without significant effects on lifespan: *CG7970*, *CG13933*, *Hsp22*, *Hsl*, *l(1)G0004* and *Fdx1* all decrease productivity; *babos* and *sov* have increased productivity early in life and decreased productivity later in life; and *piwi* has increased productivity at an early age. RNAi of *blw*, *boi*, *CG12018*, *CG14969*, *CG16868*, *CG17777*, *CG4452*, *CG5254*, *crn*, *CycE*, *Cyp4g1*, *dx*, *ND-ACP*, *pn* and *RpL22* had deleterious effects on lifespan in at least one sex and productivity in at least one week; and RNAi of *CG3071*, *sgg* and *zf30c* increased both lifespan and productivity in at least one sex and at least one week. RNAi of *dnc* had no effects on either trait.

RNAi of the remaining 23 genes had antagonistic pleiotropic effects on lifespan and/or productivity (Figures 3 and 5, Supplementary Tables 8 and 10). RNAi of *abo*, *CG13760*, *CG7971*, *fs(1)N*, *G9a*, and *Klp67A* had increased lifespan in at least one sex and decreased early productivity. RNAi of *CG3704*, *msn* and *sra* decreased lifespan in at least one sex and increased early productivity. RNAi of *Cdc7*, *CG3326*, *Dredd*, *egh* and *lama* decreased productivity but had sexually antagonistic effects on lifespan; whereas RNAi of *CG32809*, *CG4281*, *CG4554*, *DAAM*, and *wds* increased lifespan in at least one sex and decreased early and increased late productivity. RNAi of *Eip75B* decreased female lifespan and had increased early and decreased late productivity. Finally, RNAi of *capu* and *MED22* had increased early and decreased late productivity and decreased female and increased male lifespan; while RNAi of *CG13369* reduced early and increased late productivity and increased female and decreased male lifespan.

### Effects of RNAi knockdown on RNA abundance

We assessed the effectiveness of the RNAi knockdown for 16 candidate genes in female ovaries and RNAi knockdown of 12 genes in male accessory glands using quantitative PCR (qPCR) (Supplementary Tables 11, 12; Supplementary Figures 1, 2). At the individual gene level, nine of RNAi lines tested in females and six of the RNAi lines tested in males had significantly reduced expression compared to the control. However, all genes (with the exception of *abo* in accessory glands) had lower point estimates of expression in the RNAi lines relative to the control, and the combined analyses across all genes showed significant effects of gene (Supplementary Tables 11, 12). Based on the large standard errors, we infer the qPCR experiment to quantify gene expression differences between the RNAi lines and the control were underpowered, and that the number of biological samples would need to be increased to detect small changes in gene expression for some of these genes. In contrast, the phenotypic data were based on large sample sizes and are thus reliable read-outs of the effects of small differences in gene expression in reproductive tissues.

## Discussion

Natural *D. melanogaster* populations harbor considerable segregating genetic variation for lifespan, as evidenced by the large number of QTL affecting lifespan (Nuzhdin *et al.* 1997; Leips and Mackay 2000; Pasyukova *et al.* 2000; Vieira *et al.* 2000; Leips and Mackay 2002; Forbes *et al.* 2004; Wilson *et al.* 2006; Durham *et al.* 2014; Ivanov *et al.* 2015; Huang *et al.* 2020) and rapid evolution of long-lived strains by selecting for increased age of reproduction from several different geographical populations (Rose 1984; Luckinbill *et al.* 1984; Sgrò and Partridge 1999). Previously, we assessed the genetic divergence between five long-lived O lines selected for postponed senescence and five B control lines with normal lifespan (Rose 1984; Carnes *et al.* 2015), and identified 1,925 nominally significant (*P* < 10^−3^) genes based on analyses of individual variants. Here, we re-analyzed these data for signatures of local selective sweeps involving multiple variants and identified 1,071 genes in these intervals. Clearly, the genetic architecture of natural variation in lifespan is highly polygenic.

These data can be used to begin to address two fundamental questions regarding naturally occurring genetic variation for lifespan and reproductive senescence: what are the causal genes, and what is the distribution of direct and pleiotropic effects of causal variants on each trait? However, not all of the candidate genes implicated by the divergence analyses are likely to be causal, since selection will cause local linkage disequilibrium between the focal variants and closely linked variants. We chose a subset of candidate genes to evaluate with low divergence *P*-values and for which RNAi reagents with no off-target effects were publicly available. In addition, we assessed whether the difference in reproductive senescence between the O and B lines was due to maternal genotype, paternal genotype, or both. We found that late life reproductive capacity is primarily determined by the female genotype since O line females are on average more productive in weeks three and four than are B line females. However, the male genotype is also significant at weeks one, three and four, such that the productivity of O females crossed to B males is greater than that of O females crossed to O males. One plausible explanation for the paternal effect may be differences in the accessary gland products between the O and B males. Male accessory gland products evolve rapidly and have profound effects on female life history traits, increasing egg production, decreasing lifespan and reducing the probability of re-mating (Sirot *et al.* 2015). It is possible that accessory gland products of O males are not as effective in reducing female lifespan and increasing early egg production as those of B males. Thus, we chose to evaluate candidate genes that are expressed in both ovaries and accessory glands and used a *GAL4* driver that is specific to these tissues (as well as for amniosera and seminal vesicles).

Of the 57 candidate genes tested, all but *dunce* affected lifespan and/or productivity (Supplementary Table 13). RNAi of six genes increased lifespan and/or reproduction, suggesting that normal levels of expression of these genes in ovaries and accessory glands inhibit extended lifespan and/or reproduction. RNAi of *CG3071*, *sgg* and *zf30c* results in increased lifespan and productivity; RNAi of *Lztr1* and *pcx* increases lifespan with no deleterious effects on productivity; while *piwi* RNAi increases productivity with no deleterious effects on lifespan. Thus, increasing lifespan does not necessarily occur at the cost of reduced reproductive capacity (although other, unmeasured, fitness components may be affected). *piwi* and *sgg* are highly pleiotropic genes known to affect oogenesis; in addition, both genes have been independently associated with naturally occurring genetic variation in lifespan (Huang *et al.* 2020) and response to selection for delayed reproductive senescence (Luckinbill *et al.* 1984; Fabian *et al.* 2018).

Not surprisingly, RNAi of 23 genes decreased lifespan and/or productivity (Supplementary Table 13), as would be expected if normal levels of expression of these genes in ovaries and accessory glands are required for average lifespan and productivity. This suggests there may be naturally occurring variation in levels of expression of these genes in these tissues, and selection resulted in their increased expression in ovaries and accessory glands in the O lines. Regardless of whether this testable hypothesis is true, variation in nine of these genes (*blw*, *CG14969*, *CG4452*, *CG7970*, *CycE*, *Fdx1*, *Hsp22*, *l(1)G0004*, *ND-ACP*) has been independently associated with variation in lifespan (Garcia *et al.* 2017; Huang *et al.* 2020) and response to selection for delayed reproductive senescence (Remolina *et al.* 2012) in other populations.

RNAi of the remaining 27 genes in ovaries and accessory glands resulted in antagonistic pleiotropic effects that varied in their complexity (Supplementary Table 13): antagonistic pleiotropy between lifespan and reproduction; sexual antagonistic pleiotropy for lifespan with no effects on productivity; antagonistic pleiotropic effects on early and late reproduction with no effects on lifespan; sexual antagonistic pleiotropy for lifespan and antagonistic pleiotropy between lifespan and reproduction for one sex; antagonistic pleiotropy between early and late reproduction and antagonistic pleiotropy between lifespan and early reproduction; and sexual antagonistic pleiotropy for lifespan, antagonistic pleiotropy between early and late reproduction, and antagonistic pleiotropy between lifespan and reproduction. Variation in genes exhibiting antagonistic effects between female lifespan and productivity affects alternative resource allocation strategies in terms of reproduction and somatic cell maintenance. Variation in genes with sexually antagonistic effects on lifespan affects variation in alternative life history strategies in the two sexes. All sources of antagonistic pleiotropy can lead to the maintenance of genetic variation for lifespan and reproductive senescence at intermediate frequencies in natural populations, depending on the model assumptions (Levene 1953; Williams 1957; Zajitschek and Connallon 2018). Seven genes (*babos*, *capu*, *CG4554*, *CG7971*, *DAAM*, *Eip75B*, *Klp67A*) with antagonistic pleiotropic effects between the sexes and/or between lifespan and productivity were associated with variation in lifespan or response to selection for delayed reproductive senescence in independent populations (Luckinbill *et al.* 1984; Remolina *et al.* 2012; Fabian *et al.* 2018; Huang *et al.* 2019).

In addition to *piwi* and *sgg*, 10 of the candidate genes also affect oogenesis, spermatogenesis or another aspect of reproduction (*abo*, *capu*, *Cdc7*, *CycE*, *Dredd*, *egh*, *Eip75B*, *fs(1)N*, *MED22*, *sra*) (Supplementary Table 13) (Rübsam *et al.* 1998; Kozlova and Thummel 2000; Takeo *et al.* 2012; Quinlan 2013; Lu and Fuller 2015; Stephenson *et al.* 2015; Ellis and Carney 2011). Many of the candidate genes have pleiotropic effects on a large number of different molecular functions and biological processes; for these genes, it is not obvious via which of these annotations the effect on lifespan and reproduction is exerted. For example, *Dredd* affects sperm individualization, but also regulates defense against Gram-negative bacteria (Leulier *et al.* 2000; Jang *et al.* 2006); *Eip75B* affects oogenesis and also antimicrobial humoral response (Kleino *et al.* 2005), and *Cdc7* affects histone phosphorylation and eggshell chorion gene amplification (Stephenson *et al.* 2015). Genes known to affect a host of different biological processes affect lifespan and/or productivity, including (but not limited to) mRNA splicing (Herold *et al.* 2009), ATP synthesis (Di Cara *et al.* 2013), detoxification of xenobiotics (Qiu *et al.* 2012), translation (Alonso and Santarén 2006), carbohydrate phosphorylation (Gaudet *et al.* 2011), oligosaccharide biosynthetic processing (Ten Hagen *et al.* 2003), microtubule segregation (Zhang *et al.* 2007) and imaginal disc cell formation (Klebes *et al.* 2005). Finally, this study has annotated effects on lifespan and productivity for 10 computationally predicted genes (*CG13760*, *CG14969*, *CG17777*, *CG18273*, *CG32809*, *CG4281*, *CG4452*, *CG4554*, *CG5254*, *CG7970*) for which prior information on their function was lacking.

This study is the first step to ‘reverse engineer’ the genomic response to selection for delayed reproductive senescence and the accompanying correlated response of increased lifespan from variation segregating in a natural population. We have shown that an RNAi screen targeting knockdown of gene expression in reproductive tissues in both sexes for genes that exhibit genetic divergence in replicate selection lines successfully identified genes affecting lifespan and/or reproduction. Further, the effects of RNAi knockdown are consistent with predictions of evolutionary theory, in that RNAi knockdown can result in complex sexually antagonistic pleiotropic effects on lifespan and antagonistic pleiotropic effects between lifespan and productivity – such alleles would remain segregating at intermediate frequencies in nature. RNAi resulting in increased or decreased lifespan (and/or productivity) may mimic alleles remaining at intermediate frequency due to antagonistic pleiotropic effects on other fitness traits not assessed in his study. However, it is important to note that many genes for which RNAi caused decreased lifespan and/or productivity may not be direct targets of selection, but in linkage disequilibrium with the true causal loci. Several of our candidate genes are very closely linked, and only one of the genes in a high divergence interval has increased lifespan and/or productivity. For example, *Klp67A* has increased female lifespan, but not the tightly linked genes *CG4452*, *Fdx1* and *Hsp22*, which all have decreased lifespan and/or productivity; and *Nmd3* has increased female lifespan but the adjacent gene *pn* has decreased female lifespan (Supplementary Table 13). On the other hand, *fs(1)N* and *DAAM* are adjacent; as are *CG13760*, *wds* and *egh*: RNAi of all of these genes causes increased lifespan (Supplementary Table 13). Therefore, some large divergence intervals may actually be due to selection of several closely linked genes with effects in the same direction.

The genes for which RNAi causes increased lifespan in at least one sex are excellent candidates for the next step of the reverse engineering paradigm, which is to identify the actual variants that cause increased lifespan. Are there single variants in these genes that have sexually antagonistic effects on lifespan or antagonistic pleiotropic effects on productivity, or are different, closely linked variants in these genes independently causing these effects? What are the mechanisms by which these variants affect lifespan, and how do variants in different genes interact? Are their effects the same in short-lived and long-lived genetic backgrounds? Recent advances in gene editing technology will facilitate these future studies.

## References

[bib1] Alonso J., and Santarén J. F., 2006 Characterization of the *Drosophila melanogaster* ribosomal proteome. J. Proteome Res. 5: 2025–2032. 10.1021/pr0601483 16889426

[bib2] Apfeld J., and Kenyon C., 1999 Regulation of lifespan by sensory perception in *Caenorhabditis elegans*. Nature 402: 804–809. 10.1038/45544 10617200

[bib3] Ayyadevara S., Ayyadevara R., Hou S., Thaden J. J., and Shmookler Reis R. J., 2001 Genetic mapping of quantitative trait loci governing longevity of *Caenorhabditis elegans* in recombinant-inbred progeny of a Bergerac-Bo x Rc301 interstrain cross. Genetics 157: 655–666. 11156986 10.1093/genetics/157.2.655 PMC1461506

[bib4] Beekman M., Blanché H., Perola M., Hervonen A., Bezrukov V., 2013 Genome-wide linkage analysis for human longevity: Genetics of Healthy Aging Study. Aging Cell 12: 184–193. 10.1111/acel.12039 23286790 PMC3725963

[bib5] Blüher M., Kahn B. B., and Kahn C. R., 2003 Extended longevity in mice lacking the insulin receptor in adipose tissue. Science 299: 572–574. 10.1126/science.1078223 12543978

[bib6] Bodnar A. G., Ouellette M., Frolkis M., Holt S. E., Chiu C. P., 1998 Extension of life-span by introduction of telomerase into normal human cells. Science 279: 349–352. 10.1126/science.279.5349.349 9454332

[bib7] Di Cara F., Duca E., Dunbar D. R., Cagney G., and Heck M. M. S., 2013 Invadolysin, a conserved lipid-droplet-associated metalloproteinase, is required for mitochondrial function in Drosophila. J. Cell Sci. 126: 4769–4781. 10.1242/jcs.133306 23943867 PMC3795342

[bib8] Carnes M. U., Campbell T., Huang W., Butler D. G., Carbone M. A., 2015 The genomic basis of postponed senescence in *Drosophila melanogaster*. PLoS One 10: e0138569 10.1371/journal.pone.0138569 26378456 PMC4574564

[bib9] Charlesworth B., and Hughes K. A., 1996 Age-specific inbreeding depression and components of genetic variance in relation to the evolution of senescence. Proc. Natl. Acad. Sci. USA 93: 6140–6145. 10.1073/pnas.93.12.6140 8650233 PMC39203

[bib10] Clancy D. J., Gems D., Harshman L. G., Oldham S., Stocker H., 2001 Extension of life-span by loss of *Chico*, a Drosophila insulin receptor substrate protein. Science 292: 104–106. 10.1126/science.1057991 11292874

[bib11] de Boer J., Andressoo J. O., de Wit J., Huijmans J., Beems R. B., 2002 Premature aging in mice deficient in DNA repair and transcription. Science 296: 1276–1279. 10.1126/science.1070174 11950998

[bib12] Deelen J., Beekman M., Capri M., Franceschi C., and Slagboom P. E., 2013 Identifying the genomic determinants of aging and longevity in human population studies: Progress and challenges. BioEssays 35: 386–396. 10.1002/bies.201200148 23423909 PMC3633240

[bib13] Deelen J., Beekman M., Uh H.-W., Broer L., Ayers K. L., 2014 Genome-wide association meta-analysis of human longevity identifies a novel locus conferring survival beyond 90 years of age. Hum. Mol. Genet. 23: 4420–4432. 10.1093/hmg/ddu139 24688116 PMC4103672

[bib14] Defossez P. A., Lin S. J., and McNabb D. S., 2001 Sound silencing: The Sir2 protein and cellular senescence. BioEssays 23: 327–332. 10.1002/bies.1047 11268038

[bib15] Dietzl G., Chen D., Schnorrer F., Su K.-C., Barinova Y., 2007 A genome-wide transgenic RNAi library for conditional gene inactivation in Drosophila. Nature 448: 151–156. 10.1038/nature05954 17625558

[bib16] Doria G., Barattini P., Scarpi S., Puel A., Guidi L., 2004 Role of immune responsiveness and DNA repair capacity genes in ageing. Ageing Res. Rev. 3: 143–151. 10.1016/j.arr.2003.04.001 15177051

[bib17] Durham M. F., Magwire M. M., Stone E. A., and Leips J., 2014 Genome-wide analysis in Drosophila reveals age-specific effects of SNPs on fitness traits. Nat. Commun. 5: 4338 10.1038/ncomms5338 25000897

[bib18] Ellis L. L., and Carney G. E., 2011 Socially-responsive gene expression in male *Drosophila melanogaster* is influenced by the sex of the interacting partner. Genetics 187: 157–169. 10.1534/genetics.110.122754 20980240 PMC3018301

[bib19] Fabian D. K., Garschall K., Klepsatel P., Santos-Matos G., Sucena É., 2018 Evolution of longevity improves immunity in Drosophila. Evol. Lett. 2: 567–579. 10.1002/evl3.89 30564440 PMC6292704

[bib20] Felix T. M., Hughes K. A., Stone E. A., Drnevich J. M., and Leips J., 2012 Age-specific variation in immune response in *Drosophila melanogaster* has a genetic basis. Genetics 191: 989–1002. 10.1534/genetics.112.140640 22554890 PMC3389989

[bib21] Finch C. E., and Ruvkun G., 2001 The genetics of aging. Annu. Rev. Genomics Hum. Genet. 2: 435–462. 10.1146/annurev.genom.2.1.435 11701657

[bib22] Finch C. E., and Tanzi R. E., 1997 Genetics of aging. Science 278: 407–411. 10.1126/science.278.5337.407 9334291

[bib23] Forbes S. N., Valenzuela R. K., Keim P., and Service P. M., 2004 Quantitative trait loci affecting life span in replicated populations of *Drosophila melanogaster*. I. Composite interval mapping. Genetics 168: 301–311. 10.1534/genetics.103.023218 15454544 PMC1448087

[bib24] Friedman D. B., and Johnson T. E., 1988 A mutation in the *Age-1* gene in *Caenorhabditis elegans* lengthens life and reduces hermaphrodite fertility. Genetics 118: 75–86. 8608934 10.1093/genetics/118.1.75 PMC1203268

[bib25] Garcia J. F., Carbone M. A., Mackay T. F. C., and Anholt R. R. H., 2017 Regulation of Drosophila lifespan by *bellwether* promoter alleles. Sci. Rep. 7: 4109 10.1038/s41598-017-04530-x 28646164 PMC5482829

[bib26] Gaudet P., Livstone M. S., Lewis S. E., and Thomas P. D., 2011 Phylogenetic-based propagation of functional annotations within the Gene Ontology Consortium. Brief. Bioinform. 12: 449–462. 10.1093/bib/bbr042 21873635 PMC3178059

[bib27] Giannakou M. E., Goss M., Jünger M. A., Hafen E., Leevers S. J., 2004 Long-lived Drosophila with overexpressed *Dfoxo* in adult fat body. Science 305: 361 10.1126/science.1098219 15192154

[bib28] Gil E. B., Link E. M., Liu L. X., Johnson C. D., and Lees J. A., 1999 Regulation of the insulin-like developmental pathway of *Caenorhabditis elegans* by a homolog of the PTEN tumor suppressor gene. Proc. Natl. Acad. Sci. USA 96: 2925–2930. 10.1073/pnas.96.6.2925 10077613 PMC15871

[bib29] Grandison R. C., Piper M. D. W., and Partridge L., 2009 Amino-acid imbalance explains extension of lifespan by dietary restriction in Drosophila. Nature 462: 1061–1064. 10.1038/nature08619 19956092 PMC2798000

[bib30] Griswold C. M., Matthews A. L., Bewley K. E., and Mahaffey J. W., 1993 Molecular characterization and rescue of acatalasemic mutants of *Drosophila melanogaster*. Genetics 134: 781–788. 8349109 10.1093/genetics/134.3.781 PMC1205515

[bib31] Herold N., Will C. L., Wolf E., Kastner B., Urlaub H., 2009 Conservation of the protein composition and electron microscopy structure of *Drosophila melanogaster* and human spliceosomal complexes. Mol. Cell. Biol. 29: 281–301. 10.1128/MCB.01415-08 18981222 PMC2612486

[bib32] Holzenberger M., Dupont J., Ducos B., Leneuve P., Géloën A., 2003 IGF-1 receptor regulates lifespan and resistance to oxidative stress in mice. Nature 421: 182–187. 10.1038/nature01298 12483226

[bib33] Horn L., Leips J., and Starz-Gaiano M., 2014 Phagocytic ability declines with age in adult *Drosophila* hemocytes. Aging Cell 13: 719–728. 10.1111/acel.12227 24828474 PMC4116448

[bib34] Huang W., Campbell T., Carbone M. A., Jones W. E., Unselt D., 2020 Context-dependent genetic architecture of Drosophila lifespan. PLoS Biol. (in press). 10.1371/journal.pbio.3000645 PMC7077879 32134916

[bib35] Hughes K. A., and Charlesworth B., 1994 A genetic analysis of senescence in Drosophila. Nature 367: 64–66. 10.1038/367064a0 8107775

[bib36] Hwangbo D. S., Gersham B., Tu M.-P., Palmer M., and Tatar M., 2004 Drosophila *DFOXO* controls lifespan and regulates insulin Signaling in brain and fat body. Nature 429: 562–566. 10.1038/nature02549 15175753

[bib37] Ishii N., Fujii M., Hartman P. S., Tsuda M., Yasuda K., 1998 A mutation in succinate dehydrogenase cytochrome b causes oxidative stress and ageing in nematodes. Nature 394: 694–697. 10.1038/29331 9716135

[bib38] Ivanov D. K., Escott-Price V., Ziehm M., Magwire M. M., Mackay T. F. C., 2015 Longevity GWAS using the Drosophila Genetic Reference Panel. J. Gerontol. A Biol. Sci. Med. Sci. 70: 1470–1478. 10.1093/gerona/glv047 25922346 PMC4631106

[bib39] Jackson A. U., Galecki A. T., Burke D. T., and Miller R. A., 2002 Mouse loci associated with life span exhibit sex-specific and epistatic effects. J. Gerontol. A Biol. Sci. Med. Sci. 57: B9–B15. 10.1093/gerona/57.1.B9 11773201

[bib40] Jang I.-H., Chosa N., Kim S.-H., Nam H.-J., Lemaitre B., 2006 A Spätzle-processing enzyme required for Toll signaling activation in Drosophila innate immunity. Dev. Cell 10: 45–55. 10.1016/j.devcel.2005.11.013 16399077

[bib41] Kenyon C., Chang J., Gensch E., Rudner A., and Tabtiang R., 1993 A *C. Elegans* mutant that lives twice as long as wild type. Nature 366: 461–464. 10.1038/366461a0 8247153

[bib42] Kharade S. V., Mittal N., Das S. P., Sinha P., and Roy N., 2005 Mrg19 depletion increases *S. cerevisiae* lifespan by augmenting ROS defence. FEBS Lett. 579: 6809–6813. 10.1016/j.febslet.2005.11.017 16336970

[bib43] Kim S., Benguria A., Lai C.-Y., and Jazwinski S. M., 1999 Modulation of life-span by histone deacetylase genes in *Saccharomyces cerevisiae*. Mol. Biol. Cell 10: 3125–3136. 10.1091/mbc.10.10.3125 10512855 PMC25567

[bib44] Kimura K. D., Tissenbaum H. A., Liu Y., and Ruvkun G., 1997 *Daf-2*, an insulin receptor-like gene that regulates longevity and diapause in *Caenorhabditis elegans*. Science 277: 942–946. 10.1126/science.277.5328.942 9252323

[bib45] Kirkwood T. B. L., 1977 Evolution of ageing. Nature 270: 301–304. 10.1038/270301a0 593350

[bib46] Klebes A., Sustar A., Kechris K., Li H., Schubiger G., 2005 Regulation of cellular plasticity in drosophila imaginal disc cells by the Polycomb group, Trithorax group and Lama genes. Development 132: 3753–3765. 10.1242/dev.01927 16077094

[bib47] Kleino A., Valanne S., Ulvila J., Kallio J., Myllymäki H., 2005 Inhibitor of Apoptosis 2 and TAK1-Binding Protein are components of the Drosophila Imd pathway. EMBO J. 24: 3423–3434. 10.1038/sj.emboj.7600807 16163390 PMC1276168

[bib48] Kozlova T., and Thummel C. S., 2000 Steroid regulation of postembryonic development and reproduction in Drosophila. Trends Endocrinol. Metab. 11: 276–280. 10.1016/S1043-2760(00)00282-4 10920384

[bib49] Lakowski B., and Hekimi S., 1998 The genetics of caloric restriction in *Caenorhabditis elegans*. Proc. Natl. Acad. Sci. USA 95: 13091–13096. 10.1073/pnas.95.22.13091 9789046 PMC23719

[bib50] Lang D. H., Gerhard G. S., Griffith J. W., Vogler G. P., Vandenbergh D. J., 2010 Quantitative trait loci (QTL) analysis of longevity in C57bl/6j by Dba/2j (Bxd) recombinant inbred mice. Aging Clin. Exp. Res. 22: 8–19. 10.1007/BF03324809 20305363

[bib51] Leips J., and Mackay T. F. C., 2000 Quantitative trait loci for life span in *Drosophila melanogaster*: Interactions with genetic background and larval density. Genetics 155: 1773–1788. 10924473 10.1093/genetics/155.4.1773 PMC1461186

[bib52] Leips J., and Mackay T. F., 2002 The complex genetic architecture of Drosophila life span. Exp. Aging Res. 28: 361–390. 10.1080/03610730290080399 12227919

[bib53] Leulier F., Rodriguez A., Khush R. S., Abrams J. M., and Lemaitre B., 2000 The Drosophila caspase Dredd is required to resist gram-negative bacterial infection. EMBO Rep. 1: 353–358. 10.1093/embo-reports/kvd073 11269502 PMC1083747

[bib54] Levene H., 1953 Genetic equilibrium when more than one ecological niche is available. Am. Nat. 87: 331–333. 10.1086/281792

[bib55] Libert S., Zwiener J., Chu X., Vanvoorhies W., Roman G., 2007 Regulation of Drosophila life span by olfaction and food-derived odors. Science 315: 1133–1137. 10.1126/science.1136610 17272684

[bib56] Lin S.-J., Kaeberlein M., Andalis A. A., Sturtz L. A., Defossez P.-A., 2002 Calorie restriction extends *Saccharomyces cerevisiae* lifespan by increasing respiration. Nature 418: 344–348. 10.1038/nature00829 12124627

[bib57] Lin Y. J., Seroude L., and Benzer S., 1998 Extended life-span and stress resistance in the Drosophila mutant *methuselah*. Science 282: 943–946. 10.1126/science.282.5390.943 9794765

[bib58] Lithgow G. J., White T. M., Melov S., and Johnson T. E., 1995 Thermotolerance and extended life-span conferred by single-gene mutations and induced by thermal stress. Proc. Natl. Acad. Sci. USA 92: 7540–7544. 10.1073/pnas.92.16.7540 7638227 PMC41375

[bib59] Lu C., and Fuller M. T., 2015 Recruitment of mediator complex by cell type and stage-specific factors required for tissue-specific Taf dependent gene activation in an adult stem cell lineage. PLoS Genet. 11: e1005701 10.1371/journal.pgen.1005701 26624996 PMC4666660

[bib60] Luckinbill L. S., Arking R., Clare M. J., Cirocco W. C., and Buck S. A., 1984 Selection for delayed senescence in *Drosophila melanogaster*. Evolution 38: 996–1003. 10.1111/j.1558-5646.1984.tb00369.x 28555795

[bib61] Ma L., Ma J., and Kanyan X., 2015 Effect of spaceflight on the circadian rhythm, lifespan and gene expression of *Drosophila melanogaster*. PLoS One 10: e0121600 Eratum: e0139758. 10.1371/journal.pone.0121600 25798821 PMC4370389

[bib62] Magwire M. M., Yamamoto A., Carbone M. A., Roshina N. V., Symonenko A. V., 2010 Quantitative and molecular genetic analyses of mutations increasing Drosophila life span. PLoS Genet. 6: e1001037 10.1371/journal.pgen.1001037 20686706 PMC2912381

[bib63] Medawar P. B., 1952 An Unsolved Problem in Biology, Lewis, London.

[bib64] Mockett R. J., and Sohal R. S., 2006 Temperature-dependent trade-offs between longevity and fertility in the Drosophila mutant, *methuselah*. Exp. Gerontol. 41: 566–573. 10.1016/j.exger.2006.03.015 16677788

[bib65] Nuzhdin S. V., Pasyukova E. G., Dilda C. L., Zeng Z.-B., and Mackay T. F., 1997 Sex-specific quantitative trait loci affecting longevity in *Drosophila melanogaster*. Proc. Natl. Acad. Sci. USA 94: 9734–9739. 10.1073/pnas.94.18.9734 9275193 PMC23259

[bib66] Paaby A. B., Bergland A. O., Behrman E. L., and Schmidt P. S., 2014 A highly pleiotropic amino acid polymorphism in the Drosophila insulin receptor contributes to life-history adaptation. Evolution 68: 3395–3409. 10.1111/evo.12546 25319083 PMC5079517

[bib67] Paaby A. B., and Schmidt P. S., 2008 Functional significance of allelic variation at *methuselah*, an aging gene in Drosophila. PLoS One 3: e1987 10.1371/journal.pone.0001987 18414670 PMC2288678

[bib68] Paradis S., and Ruvkun G., 1998 *Caenorhabditis elegans* Akt/PKB transduces insulin receptor-like signals from AGE-1 PI3 kinase to the DAF-16 transcription factor. Genes Dev. 12: 2488–2498. 10.1101/gad.12.16.2488 9716402 PMC317081

[bib69] Parkes T. L., Elia A. J., Dickinson D., Hilliker A. J., Phillips J. P., 1998 Extension of Drosophila lifespan by overexpression of human SOD1 in motorneurons. Nat. Genet. 19: 171–174. 10.1038/534 9620775

[bib70] Pasyukova E. G., Roshina N. V., and Mackay T. F. C., 2004 *Shuttle Craft*: A candidate quantitative trait gene for Drosophila lifespan. Aging Cell 3: 297–307. 10.1111/j.1474-9728.2004.00114.x 15379853

[bib71] Pasyukova E. G., Vieira C., and Mackay T. F. C., 2000 Deficiency mapping of quantitative trait loci affecting longevity in *Drosophila melanogaster*. Genetics 156: 1129–1146. 11063689 10.1093/genetics/156.3.1129 PMC1461330

[bib72] Ping Y., Hahm E.-T., Waro G., Song Q., Vo-Ba D.-A., 2015 Linking Aβ42-induced hyperexcitability to neurodegeneration, learning and motor deficits, and a shorter lifespan in an Alzheimer’s model. PLoS Genet. 11: e1005025 10.1371/journal.pgen.1005025 25774758 PMC4361604

[bib73] Pitt J. N., and Kaeberlein M., 2015 Why is aging conserved and what can we do about it? PLoS Biol. 13: e1002131 Erratum: e1002176. 10.1371/journal.pbio.1002131 25923592 PMC4414409

[bib74] Qiu Y., Tittiger C., Wicker-Thomas C., Le Goff G., Young S., 2012 An insect-specific P450 oxidative decarbonylase for cuticular hydrocarbon biosynthesis. Proc. Natl. Acad. Sci. USA 109: 14858–14863. 10.1073/pnas.1208650109 22927409 PMC3443174

[bib75] Quinlan M. E., 2013 Direct interaction between two actin nucleators is required in Drosophila oogenesis. Development 140: 4417–4425. 10.1242/dev.097337 24089467 PMC4007717

[bib76] Remolina S. C., Chang P. L., Leips J., Nuzhdin S. V., and Hughes K. A., 2012 Genomic basis of aging and life-history evolution in *Drosophila melanogaster*. Evolution 66: 3390–3403. 10.1111/j.1558-5646.2012.01710.x 23106705 PMC4539122

[bib77] Rogina B., and Helfand S. L., 2004 *Sir2* mediates longevity in the fly through a pathway related to calorie restriction. Proc. Natl. Acad. Sci. USA 101: 15998–16003. 10.1073/pnas.0404184101 15520384 PMC528752

[bib78] Rollmann S. M., Magwire M. M., Morgan T. J., Özsoy E. D., Yamamoto A., 2006 Pleiotropic fitness effects of the *Tre1-Gr5a* region in *Drosophila melanogaster*. Nat. Genet. 38: 824–829. 10.1038/ng1823 16783380

[bib79] Rose M. R., Vu L. N., Park S. U., and Graves J. L., 1992 Selection on stress resistance increases longevity in *Drosophila melanogaster*. Exp. Gerontol. 27: 241–250. 10.1016/0531-5565(92)90048-5 1521597

[bib80] Rose M. R., 1984 Laboratory evolution of postponed senescence in *Drosophila melanogaster*. Evolution 38: 1004–1010. 10.1111/j.1558-5646.1984.tb00370.x 28555803

[bib81] Rose M. R., and Charlesworth B., 1981 a Genetics of life history in *Drosophila melanogaster*. I. Sib analysis of adult females. Genetics 97: 173–186. 6790340 10.1093/genetics/97.1.173 PMC1214382

[bib82] Rose M. R., and Charlesworth B., 1981 b Genetics of life history in *Drosophila melanogaster*. II. Exploratory selection experiments. Genetics 97: 187–196. 6790341 10.1093/genetics/97.1.187 PMC1214383

[bib83] Rübsam R., Hollmann M., Simmerl E., Lammermann U., Schäfer M. A., 1998 The *egghead* gene product influences oocyte differentiation by follicle cell-germ cell interactions in *Drosophila melanogaster*. Mech. Dev. 72: 131–140. 10.1016/S0925-4773(98)00023-9 9533964

[bib84] Sgrò C. M., and Partridge L., 1999 A delayed wave of death from reproduction in Drosophila. Science 286: 2521–2524. 10.1126/science.286.5449.2521 10617470

[bib85] Shmookler Reis R. J., Kang P., and Ayyadevara S., 2006 Quantitative trait loci define genes and pathways underlying genetic variation in longevity. Exp. Gerontol. 41: 1046–1054. 10.1016/j.exger.2006.06.047 16919411

[bib86] Shook R., 1996 Mapping quantitative trait loci affecting lifehistory traits in the nematode *Caenorhabditis elegans*. Genetics 142: 801–817. 8849889 10.1093/genetics/142.3.801 PMC1207020

[bib87] Sirot L. K., Wong A., Chapman T., and Wolfner M. F., 2015 Sexual conflict and seminal fluid proteins: A dynamic landscape of sexual interactions. Cold Spring Harb. Perspect. Biol. 7: a017533 10.1101/cshperspect.a017533 PMC4315932 25502515

[bib88] Stephenson R., Hosler M. R., Gavande N. S., Ghosh A. K., and Weake V. M., 2015 Characterization of a Drosophila ortholog of the Cdc7 kinase: A role for Cdc7 in endoreplication independent of Chiffon. J. Biol. Chem. 290: 1332–1347. 10.1074/jbc.M114.597948 25451925 PMC4340381

[bib89] Sun J., Folk D., Bradley T. J., and Tower J., 2002 Induced overexpression of mitochondrial Mn-superoxide dismutase extends the life span of adult *Drosophila melanogaster*. Genetics 161: 661–672. 12072463 10.1093/genetics/161.2.661 PMC1462135

[bib90] Takeo S., Swanson S. K., Nandanan K., Nakai Y., Aigaki T., 2012 Shaggy/glycogen synthase kinase 3β and phosphorylation of Sarah/regulator of calcineurin are essential for completion of Drosophila female meiosis. Proc. Natl. Acad. Sci. USA 109: 6382–6389. 10.1073/pnas.1120367109 22421435 PMC3340032

[bib91] Ten Hagen K. G., Tran D. T., Gerken T. A., Stein D. S., and Zhang Z., 2003 Functional characterization and expression analysis of members of the UDP-GalNAc:polypeptide N-acetylgalactosaminyltransferase family from *Drosophila melanogaster*. J. Biol. Chem. 278: 35039–35048. 10.1074/jbc.M303836200 12829714

[bib93] Tissenbaum H. A., and Ruvkun G., 1998 An insulin-like signaling pathway affects both longevity and reproduction in *Caenorhabditis elegans*. Genetics 148: 703–717. 9504918 10.1093/genetics/148.2.703 PMC1459840

[bib94] Vieira C., Pasyukova E. G., Zeng Z.-B., Hackett J. B., Lyman R. F., 2000 Genotype-environment interaction for quantitative trait loci affecting life span in *Drosophila melanogaster*. Genetics 154: 213–227. 10628982 10.1093/genetics/154.1.213 PMC1460900

[bib95] Williams G. C., 1957 Pleiotropy, natural selection, and the evolution of senescence. Evolution 11: 398–411. 10.1111/j.1558-5646.1957.tb02911.x

[bib96] Wilson R. H., Morgan T. J., and Mackay T. F. C., 2006 High-resolution mapping of quantitative trait loci affecting increased life span in *Drosophila melanogaster*. Genetics 173: 1455–1463. 10.1534/genetics.105.055111 16702433 PMC1526659

[bib97] Woodhead A. D., Merry B. J., Cao E. H., Holehan A. M., Grist E., 1985 Levels of O6-methylguanine acceptor protein in tissues of rats and their relationship to carcinogenicity and aging. J. Natl. Cancer Inst. 75: 1141–1145. 3865014

[bib98] Yu C. E., Oshima J., Fu Y. H., Wijsman E. M., Hisama F., 1996 Positional cloning of the Werner’s Syndrome gene. Science 272: 258–262. 10.1126/science.272.5259.258 8602509

[bib99] Zajitschek F., and Connallon T., 2018 Antagonistic pleiotropy in species with separate sexes, and the maintenance of genetic variation in life‐history traits and fitness. Evolution 72: 1306–1316. 10.1111/evo.13493 29667189

[bib100] Zerofsky M., Harel E., Silverman N., and Tatar M., 2005 Aging of the innate immune response in *Drosophila melanogaster*. Aging Cell 4: 103–108. 10.1111/j.1474-9728.2005.00147.x 15771614

[bib101] Zhang D., Rogers G. C., Buster D. W., and Sharp D. J., 2007 Three microtubule severing enzymes contribute to the ‘pacman-flux’ machinery that moves chromosomes. J. Cell Biol. 177: 231–242. 10.1083/jcb.200612011 17452528 PMC2064132

[bib102] Zwaan B., Bijlsma R., and Hoekstra R. F., 1995 Artificial selection for developmental time in *Drosophila melanogaster* in relation to the evolution of aging: Direct and correlated responses. Evolution 49: 635–648. 10.1111/j.1558-5646.1995.tb02300.x 28565147

